# Editorial

**Published:** 1872-10

**Authors:** 


### Editorial.

The announcement of the proposed publication of "The Chicago Medical Register and Directory," will doubtless be very gratifying to the majority of our readers, indeed to all who have the true interests of the profession at heart. The work is capable of becoming most useful, not only to the profession, but to the public at large, and should eventually be made the standard of reference regarding the status of any institution, and of every member of the Medical profession. For the satisfactory accomplishment of this task, however, peculiar qualifications are demanded, and we are glad to perceive that the Editors have apparently endeavored to secure these, either in themselves or in their committee of revision.

The work proposes to indicate "The Hospitals, Infirmaries,

Dispensaries, Charitable Institutions and Asylums of the entire State of Illinois, also the Medical and Scientific Societies and Associations, with full lists of membership. It will also contain announcements of the Schools of Medicine and Pharmacy, etc."

"There will be given a list of Physicians in good standing in Chicago and vicinity, and in the larger towns in the State, with residence, office, and office hours; also a list of Dentists who are regularly graduated as D.D.S. or M.D., or who are members of a Dental Society; also a list of Veterinary Surgeons who are regularly educated," etc., etc.

The scheme is comprehensive, although in one or two points it might be made even more so with advantage.

We think it would be well to add to the name of each physician that of the college from which he graduated, and also the date of such graduation. The JOURNAL has already called the attention of the profession to the fact, that the country was overrun by a horde of charlatans holding purchased diplomas, and also become one of the initiators of the movement which resulted in the suppression of one of the worst of these diploma-factories, by legislative enactment in Pennsylvania. Our own city and State have their full quota of these self-styled Doctors, against whom the Editors of a Medical Register should exercise especial discrimination. Europe too has disgorged a swarm of medical (as well as other) impostors, into our midst who are not quite so easy of detection. In view of which fact we think it would have been wise for the Editors to have added to their Committee of Revision the name of some one of our more prominent German physicians, whose advice would prove invaluable to them in the prosecution of their investigations; of these, there are several well qualified for the task, which we fear may be imperfectly performed in this department without such co-operation. It is to be hoped that the Editors will also add to their work a list of Apothecaries, graduates in Pharmacy, or who are members of Pharmaceutical associations, that physicians and the general public may be able to discriminate between those who are and those who are not entitled to patronage and encouragement.

Let no fear of making invidious distinctions, and no mistaken charity, deter the projectors of this work from excluding rigidly from its pages the names of any and all, no matter how prominent or popular, who may not be in the fullest sense of the expression "regular and in good standing in the profession."

Let them remember that true charity must discriminate carefully, lest in palliating the delinquencies of individuals, it bring odium upon an entire profession, and great detriment to the public.

The work in question will take rank in one of two categories; either as a reliable standard of reference upon which the profession first, and, secondly, the public, will rely for accurate information, or as a compilation of excerpts from, and subsidiary to the City Directory, of no more value than a Report of the Board of Health.

Should it merit position in the first class, it will become a permanent institution, and if not perfect at first, will, like good wine, improve with age. If, however, it should fall into the second class, like the victims of cholera infantum, it will scarcely survive its first year.

Of the gentlemen who have undertaken this work, our notice is a sufficient indication of our high opinion; they have the capacity, and we believe the intention, to make this work what its most earnest advocates could desire it to be. But unfortunately they are "*good fellows*," and we fear too kind-hearted to be as strict as they ought to be. We trust they will harden their hearts, and remember "*the greatest good to the greatest number*." H.

***National Medical Library.***

The attention of our readers is especially directed to the subjoined notice of Dr. Billings, Librarian, Surgeon General's Office, to which it may perhaps be desirable to prefix a word in explanation. The Library attached to the Surgeon General's Office at Washington, and for which Dr. B. desires the journals indicated below, is by no means exclusively for the use and benefit of the medical officers of the army; but is intended to be a National Medical Library, open to the profession and the public at large, for study and for reference. The Librarian of Congress has relinquished the Medical Department of that library to the one in question, and it has already become the most valuable source of reference in the country.

Many books, pamphlets, catalogues, odd numbers of journals, etc., which are mere literary rubbish to their possessors, could be utilized here, and become valuable to the profession.

Contributors to this National Medical Library, in reality, enrich their profession, and thus indirectly will reap the reward of their generosity. H.

---

***Wanted***—For the Library of the Surgeon General's Office, at Washington, D. C.

To complete the files of American Medical periodicals, in the above Library, the following are wanted:

Illinois Medical and Surgical Journal, Chicago, 1844-45. Wanted, No. 10, vol. I.; Nos. 6, 8, 10, 11 and 12, of vol. II.

Illinois and Indiana Medical and Surgical Journal, Chicago, 1846-47. Wanted, Nos. 3, 4, 5 and 6, vol. I.

Northwestern Medical and Surgical Journal. Wanted, No. 1, vol. II, (1849); No. 9, vol. XI, whole series; Vol. XII, whole series.

Western Medico-Chirurgical Journal, Keokuk, Iowa, 1852-53. Wanted, all or any of it.

St. Louis Medical and Surgical Journal. Wanted, vols. I and II, 1843-44.

As it is desired to make this Library a complete record of American Medicine, old Medical pamphlets of all kinds, Lectures, Addresses, Reports, Announcements of Medical Colleges, etc., are desired.

For any of the above, a fair price will be paid, or valuable exchanges will be furnished.

Pamphlets and Journals for the Library may be forwarded by mail, post free, if addressed to the Surgeon General, U. S. A., Washington, D. C., and marked in one corner "For the Library."

J. S. BILLINGS,
*Ass't Surg. U. S. A. and Librarian S. G. O.*

---

***Rush Medical College.***

The introductory exercises of the opening of the Thirtieth Annual Course of Lectures of the Rush Medical College will be held in the amphitheatre of the temporary college building, at the south-west corner of Eighteenth and Arnold streets, Chicago, at 7:30 P. M., Wednesday, October 2d, 1872. The Introductory will be delivered by J. Adams Allen, M.D., LL.D., Professor of Principles and Practice of Medicine. The unexpectedly large number of matriculants at the present writing, promises a large class for the coming session.

The Regular Lectures will commence on the morning of October 3d, and continue twenty weeks. As there will be but one Introductory, students are reminded that their interests will be served by their attendance at the opening exercises.

J. H. ETHERIDGE,
*Assistant Secretary Rush Medical College Faculty,*
CHICAGO, Sept. 18, 1872. 603 *Michigan Avenue.*

